# Quality control, modeling, and visualization of CRISPR screens with MAGeCK-VISPR

**DOI:** 10.1186/s13059-015-0843-6

**Published:** 2015-12-16

**Authors:** Wei Li, Johannes Köster, Han Xu, Chen-Hao Chen, Tengfei Xiao, Jun S. Liu, Myles Brown, X. Shirley Liu

**Affiliations:** Department of Biostatistics and Computational Biology, Dana-Farber Cancer Institute, Harvard T.H. Chan School of Public Health, Boston, MA 02215 USA; Center for Functional Cancer Epigenetics, Dana-Farber Cancer Institute, Boston, MA 02215 USA; Division of Molecular and Cellular Oncology, Department of Medical Oncology, Dana-Farber Cancer Institute, Boston, MA 02215 USA; Broad Institute of MIT and Harvard, 7 Cambridge Center, Cambridge, MA 02142 USA; Department of Statistics, Harvard University, Science Center 715, 1 Oxford Street, Cambridge, MA 02138 USA; Department of Medicine, Brigham and Women’s Hospital and Harvard Medical School, Boston, MA 02215 USA; School of Life Science and Technology, Tongji University, Shanghai, 200092 China

**Keywords:** CRISPR/Cas9, Screening, Maximum likelihood, Expectation-Maximization, Negative binomial, Data-driven documents, D3, Visualization, Quality control

## Abstract

High-throughput CRISPR screens have shown great promise in functional genomics. We present MAGeCK-VISPR, a comprehensive quality control (QC), analysis, and visualization workflow for CRISPR screens. MAGeCK-VISPR defines a set of QC measures to assess the quality of an experiment, and includes a maximum-likelihood algorithm to call essential genes simultaneously under multiple conditions. The algorithm uses a generalized linear model to deconvolute different effects, and employs expectation-maximization to iteratively estimate sgRNA knockout efficiency and gene essentiality. MAGeCK-VISPR also includes VISPR, a framework for the interactive visualization and exploration of QC and analysis results. MAGeCK-VISPR is freely available at http://bitbucket.org/liulab/mageck-vispr.

**Electronic supplementary material:**

The online version of this article (doi:10.1186/s13059-015-0843-6) contains supplementary material, which is available to authorized users.

## Background

The clustered regularly interspaced short palindromic repeats (CRISPR)/Cas9 system is a powerful genetic engineering technique, allowing direct modifications of genomic loci in most model organisms in a cost-effective way. Based on this system, the recent development of high-throughput CRISPR screening technology has shown great promise in functional genomics, allowing researchers to systematically identify genes associated with various phenotypes [1–4]. CRISPR screens can be performed by either direct knockout of genes using CRISPR/Cas9 [1, 2], or perturbing gene expressions using CRISPR and a dead-Cas9 (dCas9) fused with activation or repression effectors [5, 6].

While CRISPR screening is a powerful technique, it creates computational challenges that include: (1) how to evaluate the data quality; (2) how to identify gene or pathway hits from the screens and assess their statistical significance; and (3) how to visualize and explore the screening results efficiently. Until now, a comprehensive quality control (QC), data analysis, and visualization method for CRISPR screen was not available. Several algorithms are developed for screening analysis on microarray or high-throughput sequencing data, such as RIGER [7], RSA [8], HitSelect [9], as well as the MAGeCK algorithm we previously developed [10]. These algorithms are designed based on a comparison of two conditions, although many screens are conducted simultaneously across several time points, under many treatment conditions or over many cell lines. In addition, these algorithms do not consider the knockout efficiency of single guide RNAs (sgRNA) on target genes. The knockout efficiency is the ability of a sgRNA to induce cutting events that lead to the knockout of the targeted gene. It is influenced by sgRNA sequence content [11], chromatin accessibility and exon position of the targeting gene [12], and so on.

**Fig. 1:**
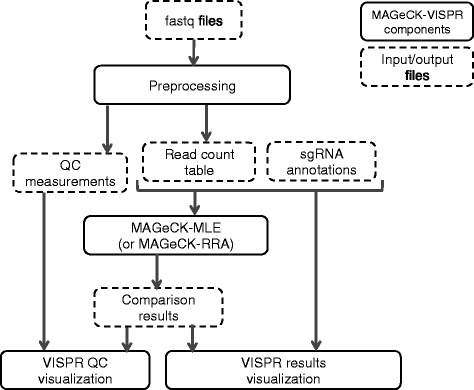
An overview of the MAGeCK-VISPR workflow. Given FASTQ files and an sgRNA design, the workflow employs several preprocessing steps, including using FastQC and MAGeCK to collect quality control metrics and calculate per-sgRNA read counts. Then, either MAGeCK-RRA or MAGeCK-MLE is used to determine essential genes under user-defined conditions. Finally, results are composed for visualization and interactive exploration in VISPR

In this study, we present MAGeCK-VISPR to overcome the computational challenges of CRISPR screens. MAGeCK-VISPR (1) defines a set of QC measurements and (2) extends the MAGeCK algorithm by a maximum likelihood estimation method (MAGeCK-MLE) to call essential genes under multiple conditions while considering sgRNA knockout efficiency. Further, MAGeCK-VISPR (3) provides a web-based visualization framework (VISPR) for interactive exploration of CRISPR screen quality control and analysis results. MAGeCK-VISPR employs a Snakemake [13] workflow to combine MAGeCK and VISPR in a scalable and reproducible way (Fig. 1).

## Results and discussion

### Quality control measurements for CRISPR screening experiments

**Fig. 2:**
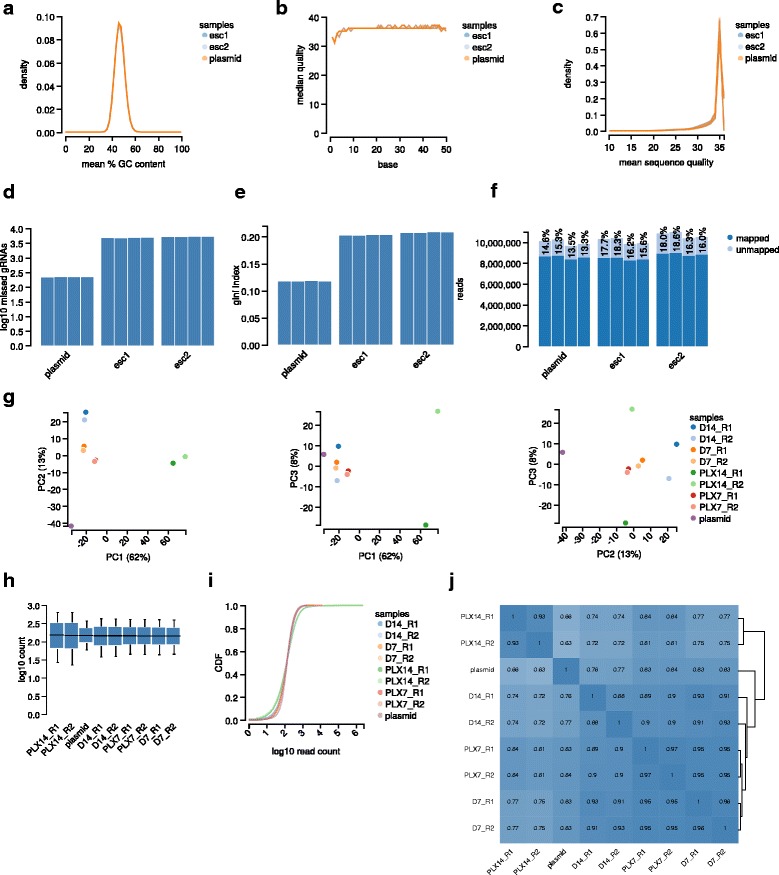
The quality control (QC) view of VISPR, the visualization framework of MAGeCK-VISPR. The measurements include the distribution of GC content (**a**), median base quality (**b**), the distribution of mean sequence quality (**c**), the number of zero-count sgRNAs (**d**), Gini-index (**e**), total number of reads and the percentage of mapped reads (**f**), Principle Component Analysis (PCA) plot (**g**), normalized read count distribution (**h**, **i**), and pairwise sample correlations (**j**). Shown results are from ESC (**a**-**f**) and melanoma dataset (**g**-**j**)

Apart from the determination of essential genes with MAGeCK, a central purpose of MAGeCK-VISPR is to collect quality control (QC) measurements at various levels (http://www.bioinformatics.babraham.ac.uk/projects/fastqc). The proposed measurements (Table 1) can be divided into four categories: sequence level, read count level, sample level, and gene level (Fig. 2).

**Table 1:** Quality control (QC) measures from MAGeCK-VISPR

QC term	Description	Expected
GC content	GC content distribution of the sequencing reads	Similar distribution for all samples from same library
Base quality	Base quality distribution of the sequencing reads	Single-peak distribution with median base quality at least 25
Sequencing reads	Total number of sequencing reads	Varies depending on sequencing platform
Mapped reads	Total number of reads mapped to the sgRNA library	300 * (number of sgRNAs)
% Mapped reads	Percentage of mapped reads to the total number of sequencing reads	At least 65 %
Zero sgRNAs	Number of sgRNAs with zero read counts	At most 1 % of total sgRNAs
Gini index	Gini index of log-scaled read count distributions	At most 0.1 for plasmid or initial state samples, and at most 0.2 for negative selection samples
Sample correlation	Pearson correlation coefficient between samples	At least 0.8 for replicates
Correlation clustering or PCA clustering	Hierarchical clustering of samples or first three PCA components	Samples with similar conditions should cluster together
Ribosomal gene selection	Negative selection enrichment statistics of ribosomal genes	Significant *P* values (<0.001) for ribosomal subunit (GO:0044391) in negative selection experiments

Sequence level QC measurements aim to detect problems with the sequencing, similar as in other next-generation sequencing (NGS) experiments. Two measurements are reported: sample GC content distribution (Fig. 2a) and the base quality distribution of sequencing reads (Fig. 2b, c). Ideally, sequencing reads should have reasonable base qualities (median value >25), and samples from the same experiment should have similar GC content distributions.

The second level of QC measurements is based on the sgRNA read counts collected from MAGeCK. Raw sequencing reads are first mapped to sgRNA sequences in the library with no mismatches tolerated. After that, the number of sequencing reads, mapped reads (and thereof the percentage of mapped reads), sgRNAs with zero read count, and the Gini index of read count distribution are reported for each sample (Fig. 2d-f). The percentage of mapped reads is a good indicator of sample quality, and low mappability could be due to sequencing error, oligonucleotide synthesis error, or sample contamination. Good statistical power of downstream analysis relies on sufficient reads (preferably over 300 reads) for each sgRNA, with low number of zero-count sgRNAs in the plasmid library or early time points. Gini index, a common measure of income inequality in economics, can measure the evenness of sgRNA read counts [14]. It is perfectly normal for later time points in positive selection experiments to have higher Gini index since a few surviving clones (a few sgRNA with extreme high counts) could dominate the final pool while most of the other cells die (more sgRNAs with zero-count). In contrast, high Gini index in plasmid library, in early time points, or in negative selection experiments may indicate CRISPR oligonucleotide synthesis unevenness, low viral transfection efficiency, and over selection, respectively.

Sample level QC (Fig. 2g-j) checks the consistency between samples. MAGeCK-VISPR reports the distributions of normalized read counts by box plots and cumulative distribution functions. It also calculates pairwise Pearson correlations of sample log read counts, and draws the samples on the first three components of a Principle Component Analysis (PCA). Biological replicates or samples with similar conditions should have similar read count distributions and higher correlations, and appear closer to each other in the PCA plot. PCA plots can also identify potential batch effects if the screens are conducted under different batches.

Finally, gene level QC determines the extent of negative selection in the screens. Since knocking out ribosomal genes lead to a strong negative selection phenotype [1, 2], the significance of negative selection on ribosomal genes can be evaluated in MAGeCK-VISPR by Gene Ontology (GO) enrichment analysis using GOrilla [15]. A working negative selection experiment should have a significant *P* value (<0.001), although many good experiments could have much smaller *P* values (<1e-10, see Section A of Additional file 1).

### Calling essential genes under multiple conditions with MAGeCK-MLE

MAGeCK-VISPR includes a new algorithm, ‘MAGeCK-MLE’, to estimate the essentiality of genes in various screening conditions using a maximum likelihood estimation (MLE) approach. Compared with the original MAGeCK algorithm using Robust Rank Aggregation (‘MAGeCK-RRA’) that can only compare samples between two conditions, MAGeCK-MLE is able to model complex experimental designs. Furthermore, MAGeCK-MLE explicitly models the sgRNA knockout efficiency, which may vary depending on different sequence contents and chromatin structures [11, 12]. In MAGeCK-MLE, the read count of a sgRNA *i* targeting gene *g* in sample *j* is modeled as a Negative Binomial (NB) random variable. The mean of the NB distribution (*μ*_*ij*_) is dependent on three factors: the sequencing depth of sample *j* (*s*_*j*_), the knockout efficiency of sgRNA *i*, and a linear combination of the effects in different conditions (that is, different drug treatments) on gene *g*. If sgRNA *i* knocks out target gene *g* efficiently, then *μ*_*ij*_ is modeled as:

$$ {\mu}_{ij}={s}_j\  exp\left({\beta}_{i0}+{\displaystyle \sum_r}{d}_{jr}{\beta}_{gr}\right) $$

The effects of *r* different conditions are represented as the score ‘*β*_*gr*_’, a measurement of gene selections similar to the term of ‘log fold change’ in differential expression analysis. The presence or absence of each condition on each sample is encoded into binary elements of the *design matrix d*_*jr*_, and can be obtained from experiment designs. ‘*β*’ scores reflect the extent of selection in each condition: *β*_*gr*_ >0 (or <0) means *g* is positively (or negatively) selected in condition *r. μ*_*ij*_ is also dependent on *β*_*i*0_, the initial sgRNA abundance which is usually measured in plasmid or the day 0 of the experiment.

The values of *β*, together with the information whether an sgRNA is efficient, can be estimated by maximizing the joint log-likelihood of observing all sgRNA read counts of *g* on all different samples, and are optimized using an Expectation-Maximization (EM) algorithm. In the EM algorithm, MAGeCK-MLE iteratively determines the knockout efficiency of each sgRNA based on the current estimation of ‘*β*’ scores (the E step), and uses the updated knockout efficiency information to re-calculate ‘*β*’ scores (the M step). By examining the patterns of read counts of each sgRNA across all samples, the EM algorithm minimizes the effect of inefficient sgRNAs. A detailed description of the method is presented in the Methods section.

**Fig. 3:**
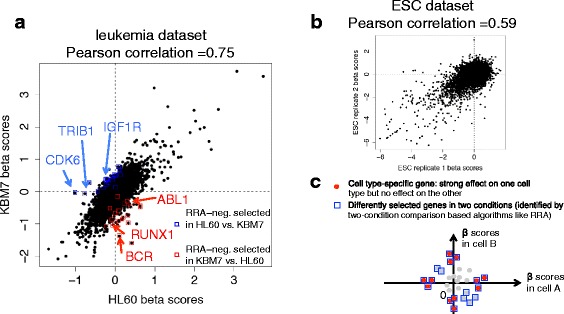
The gene essentiality scores (*β* scores) reported from MAGeCK-MLE on two conditions. **a**, **b** the *β* scores of two leukemia cell lines in the leukemia dataset (**a**), and two biological replicates of mouse ESC cells in the ESC dataset (**b**). In (**a**), some well-known driver genes and cell type-specific genes are also labeled. These genes may play distinct roles in two different leukemia subtypes (HL60: acute myeloid leukemia; KBM7: chronic myeloid leukemia), including CDK6 and TRIB1 for HL60, and RUNX1 in KBM7. CDK6 is required in AML growth [17] and TRIB1 over-expression is observed in AML patients compared with CML patients [18]. On the other hand, the frequent RUNX1 loss-of-function mutations are observed in CML to AML transformations [19]. **c** An illustration of differentially selected genes identified by two-condition comparison algorithms (like RRA, blue rectangles). MAGeCK-MLE can further distinguish cell type-specific genes (red dots) from other genes. Cell type-specific genes are genes having no essentiality in one condition but strong essentiality in the other, and are usually more biologically interesting

**Fig. 4:**
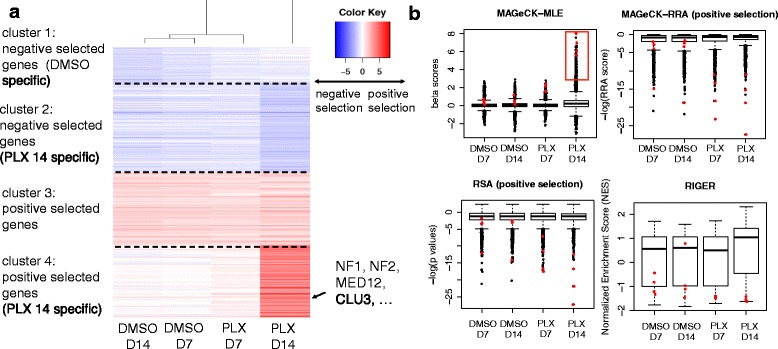
The *β* scores of MAGeCK-MLE on the melanoma knockout dataset. **a** A k-means clustering view of *β* scores of all conditions from top selected genes (k = 4). Only genes with the highest or lowest 1 % *β* scores in DMSO or PLX 14-day treatment conditions are shown. **b** The distribution of scores across four conditions using different algorithms. The red rectangle in MAGeCK-MLE indicates genes in cluster 4 in Fig. 4a, or genes that are strongly positively selected in PLX 14-day condition. Some validated genes in the original study are marked as red dots, including NF1, NF2, MED12, and CUL3

**Fig. 5:**
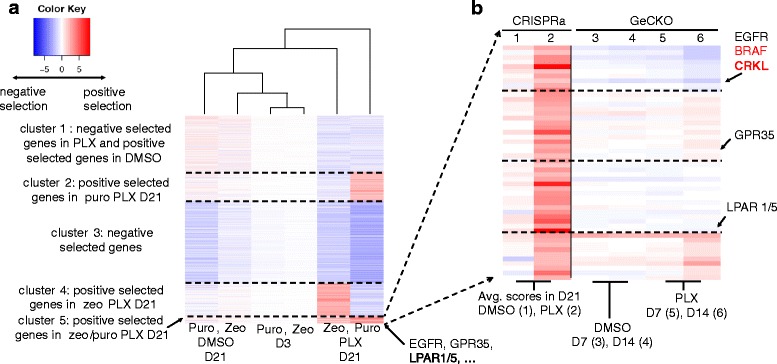
The *β* scores of MAGeCK-MLE on the melanoma activation dataset. **a** A k-means clustering view of *β* scores of all conditions from top selected genes (k = 5). Only genes with the highest or lowest 1 % *β* scores in DMSO or PLX 21-day treatment conditions are shown. **b** the average *β* scores of genes in cluster 5 of A (consistently positive selected genes in both zeocin and puromycin conditions), as well as the *β* scores of these genes in melanoma knockout dataset. Similar to (**a**), the k-means clustering algorithm is applied to the selected genes (k = 4)

We tested MAGeCK algorithms on four public datasets. The first two datasets (the ‘ESC’ and ‘leukemia’ dataset) correspond to negative selection experiments on mouse embryonic stem cells (ESCs) and two human leukemia cell lines (KBM7 and HL-60), respectively (Fig. 3a and b) [1, 4]. In both datasets, cells were grown with their natural growing condition and negative selections occurred in cells after CRISPR/Cas9 is activated. The other two datasets (‘melanoma’ knockout and activation dataset) are different CRISPR screens on the human melanoma cell line A375 that harbors a BRAF V600E mutation (Figs. 4 and 5). The cells were treated with BRAF inhibitor vemurafenib (PLX) or dimethyl sulfoxide (DMSO) control, and screened either with GeCKO [2] or with CRISPR/dCas9 Synergistic Activation Mediator (SAM) libraries [5]. These two datasets include multiple experimental conditions that are difficult to compare directly using the original MAGeCK-RRA algorithm. In the melanoma knockout dataset, cells were under 7-day or 14-day selection [2]. In the melanoma activation dataset, two different drugs (puromycin and zeocin) were used to select cells with lentiviral infection, and both DMSO and PLX treatments were profiled under 3-day or 21-day selection [5].

In two-condition comparisons, MAGeCK-MLE gives similar results with existing methods such as MAGeCK-RRA, RSA, and RIGER. All the algorithms identified genes that are commonly essential to different cell types [16], as well as known positively selected genes in PLX treated conditions in two melanoma datasets (Fig. 3; also see Section A and B of Additional file 1). In the leukemia dataset, two-condition comparison algorithms (like MAGeCK-RRA) identified genes that are differentially selected in two cell lines by a direct comparison of HL60 and KBM7 (Fig. 3a) [10]. However, not all of these genes are equally biologically interesting, as MAGeCK-MLE further distinguished them into two groups: genes having little effect in one (β scores close to zero) but strong selection effect in the other cell line (large absolute β scores), and genes having weak and opposite effects in two cell lines (Fig. 3c). The first group of genes are often more biologically interesting as they are cell type-specific genes. This includes some well-known driver genes (like BCR in KBM7) as well as genes that may be functional in only one cell type: CDK6 and TRIB1 in HL60 [17, 18], and RUNX1 in KBM7 [19].

One of the advantages of MAGeCK-MLE over other methods is that it enables accurate comparisons of gene essentialities across multiple conditions and experiments in one run (Fig. 4 and Section C of Additional file 1). In the melanoma knockout dataset, a k-means clustering of the β scores of top selected genes demonstrated that these genes have various essentialities across conditions (Fig. 4a). Some of the genes are universally positively or negatively selected in all conditions (cluster 3), while others have different essentiality across different conditions (clusters 1, 2, and 4). Genes in cluster 4 are particularly interesting as they show strong positive selection in 14-day PLX treated condition. Indeed, genes whose knockout leads to strong positive selection in PLX-treated cells are in cluster 4, including NF1, NF2, MED12, CUL3 [2]. In contrast, the k-means clusters of measurements from other algorithms did not reveal the strong effect of genes in cluster 4 (Section C of Additional file 1). This is because their score distributions are similar across different conditions (Fig. 4b), and do not reflect the fact that the one condition (PLX 14-day treatment) induces much stronger positive selection than other conditions [2]. This is partly because MAGeCK-RRA, RIGER, and RSA all use a rank-based method to compare sgRNA between two conditions, which may lose quantitative information.

Another example of using MAGeCK-MLE on multiple conditions is demonstrated in the melanoma activation dataset, where cells underwent different selection methods (using puromycin or zeocin), drug treatments (DMSO or PLX), and durations (3-day or 21-day treatment) (Fig. 5). Similar to the melanoma knockout dataset, we performed k-means clustering of the top-selected gene β scores. Many positively selected genes are dependent on the selection method, which might not be biologically interesting. For example, genes in clusters 2 and 4 correspond to positively selected genes that are specific to puromycin or zeocin selection, respectively. A small set of genes (cluster 5) are consistently selected in both zeocin and puromycin, including genes that are validated in the original study, for example, EGFR, GPR35, LPAR1/5 [5]. We further examined the genes in cluster 5 (Fig. 5b), and focused on genes positively selected in the CRISPR activation experiment but strongly negatively selected in the knockout experiment. These genes include EGFR and BRAF, two known kinases that drive melanoma progression and PLX resistance [20, 21], and CRKL, a protein kinase that activates RAS and JUN pathway. CRKL amplification is reported to lead to drug resistance against EGFR inhibitors by activating EGFR downstream pathways [22], implying its potential role in PLX drug resistance.

### Visualization of QC measurements and gene essentiality with VISPR

VISPR (VISualization of crisPR screens) is a web-based frontend for interactive visualization of CRISPR screen QC and comparison results. Interactive access is provided by an HTML5 based browser interface, while visualizations are realized with Vega [23], a declarative visualization grammar on top of Data-Driven Documents (D3) [24]. VISPR provides three types of views for interactive exploration of CRISPR screening: a quality control view, a result view, and an experiment comparison view. The quality control view shows the QC measurements described before (Fig. 2).

**Fig. 6:**
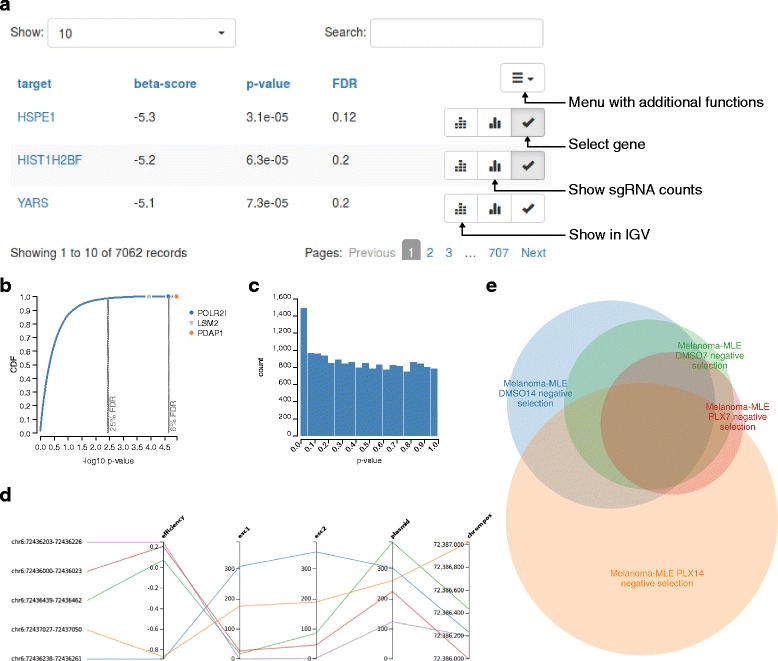
The result and comparison views of VISPR. The result view includes a gene comparison table (**a**), the distribution of *P* values as CDF (**b**) and histogram (**c**), and the normalized sgRNA counts in all samples of selected genes along with chromosome positions and predicted efficiency (**d**). The comparison view of VISPR (**e**) displays the overlap between significant genes under different selectable conditions and experiments as an Euler diagram. Shown results are from ESC (**a**, **d**) and melanoma dataset (**b**, **c**, **e**)

In the result view, screening results can be interactively explored. It contains a table showing the comparison results of each gene (Fig. 6a). The table can be sorted by different columns and filtered (from ‘Search’) via gene names or regular expressions. Further, the distribution of *P* values is displayed as cumulative distribution function (CDF) (Fig. 6b) and as a histogram (Fig. 6c). For each gene, the normalized sgRNA counts in all samples can be displayed in a parallel coordinate visualization (Fig. 6d). If available, knockout efficiency predictions [11] and gene coordinates of each sgRNA are displayed as separate axes. Axes can be reordered or toggled on or off, and sgRNAs can be highlighted by selecting ranges on each axis. Genes selected in the table are highlighted in the CDF, allowing to assess their occurrence within the *P* value distribution of all genes.

VISPR provides various ways to further explore the analysis results. Individual genes can be viewed in Ensembl [25] and IGV [26]. Selected genes can be visualized in terms of their interaction network and function via GeneMANIA [27]. Functional analysis can be performed with GOrilla [15], an online Gene Ontology (GO) enrichment analysis tool. GOrilla takes a ranked list of genes (here based on the *P* values reported by MAGeCK) to perform a threshold-free enrichment analysis. The resulting GO term enrichments can be further used for gene-level quality control.

The comparison view of VISPR can compare different experiments by visualizing the common and exclusive significant genes via Euler diagrams (Fig. 6e). Clicking on segments of the Euler diagram opens the result views of the corresponding experiments. For example, clicking on the intersection between two experiments will open ‘restricted’ result views for each experiment, where only the common significant genes are displayed. These views provide the same features as the unconstrained result views described above. However, in this case, GO enrichment analysis with GOrilla is performed with the shown genes (that is, the genes from the intersection) as foreground and the other genes of the experiment as background.

The visualizations displayed in VISPR can be downloaded as publication-ready SVG files. In addition, a command line interface is provided to store visualizations as Vega specifications. This format allows users to modify and style the output of VISPR programmatically.

### Implementation of the MAGeCK-VISPR workflow with Snakemake

We implemented the MAGeCK-VISPR workflow with the workflow management system Snakemake [13], allowing an automatic execution of some or all of the MAGeCK-VISPR functions: quality control, essential gene analysis, and visualization. Choosing a workflow management system like Snakemake has several advantages. First, the workflow steps can be automatically parallelized and executed on workstations, servers, and compute clusters without the modification of the workflow. Second, Snakemake tracks metadata (like creation date, input, and log files) for all generated result and intermediate files. This way, used data, methods, and parameters are documented comprehensively for each analysis (also called data provenance), an important requirement of reproducible science. MAGeCK-VISPR provides a command line interface to initialize the workflow in a given work directory. This installs the workflow definition as a so-called *Snakefile*, along with a configuration file and documentation. The configuration file is used to define locations of raw data and additional parameters for MAGeCK-VISPR. Once configured, the Snakefile can be executed with Snakemake. Since the Snakefile is installed into the given work directory, it can be easily modified or extended by the user.

We provide all components of the workflow as Conda packages [28], such that MAGeCK-VISPR can be installed with a single command. Optionally, the Conda package manager can create isolated environments for the workflow to, for example, freeze or compare different software versions or publish snapshots of a MAGeCK-VISPR workflow instance along with all data and used software. This further increases the reproducibility of the generated results.

## Conclusion

The recently developed CRISPR screening is a powerful technology in functional studies with different foci, including tumor progression and metastasis [29], drug resistance [3], immune response [30], and stem cell differentiation [4]. To our knowledge, MAGeCK-VISPR is the first comprehensive pipeline developed for quality control, analysis, and visualization of CRISPR screens, and highlights new features compared with existing screening analysis algorithms. For example, a typical CRISPR screening experiment usually includes complex designs that are difficult to analyze using existing algorithms, as they are all designed for two-condition comparisons. To address this challenge, MAGeCK-VISPR uses a maximum likelihood approach to estimate the effect of different conditions using a generalized linear model (GLM). It also incorporates sgRNA knockout efficiency information by using a probabilistic mixture model. We demonstrated that MAGeCK-MLE provides additional insights into cell type-specific essential genes and is able to compare gene essentiality scores across conditions or even experiments. Also, MAGeCK-VISPR is able to handle screens of different types including CRISPR knockout and CRISPR activation screens, and can be potentially applied to high-throughput sequencing datasets of traditional RNA interference (RNAi) screens.

The MAGeCK-MLE approach is able to estimate sgRNA knockout efficiencies from CRISPR screens besides gene essentiality. We previously reported that sequence-specific features learned from CRISPR screening data helped the design of efficient sgRNAs [11]. With more CRISPR screen data becoming available, the algorithm will help us identify sgRNAs with the best behavior and learn patterns of ‘good’ sgRNAs. The information will further guide the design of optimized sgRNAs for CRISPR screens and individual gene knockouts.

One potential limitation of MAGeCK-MLE is that its EM algorithm uses an iterative process involving matrix operations, making it slower than our previous MAGeCK RRA method and other competing algorithms. Future approaches to speed up MAGeCK-MLE include improving parametric tests for *P* value estimation (instead of using permutation) and implementing the algorithm in Cython instead of Python. Another potential limitation of MAGeCK-VISPR on the quality control assessment is that the current QC thresholds for ‘successful’ experiments are determined heuristically due to limited number of publicly available CRISPR screening datasets. We and other researchers have previously reported that bigger collections of ChIP-seq datasets provide better criteria on ChIP-seq quality control [31, 32]. As more public CRISPR screening datasets become available, the QC metrics (and other parts of MAGeCK-VISPR) can be further refined.

As CRISPR screens become more popular, complications in the data such as batch effects will be unavoidable which need proper correction for meaningful downstream analysis. Existing batch removal algorithms, including ComBat [33] and RUVseq [34], have been widely used to remove batch effects in gene expression analysis. In the future, these algorithms can be integrated into MAGeCK-VISPR pipeline. After that, MAGeCK-VISPR will be able to identify cancer- and disease-specific essential genes by a direct comparison between different datasets or experiments, providing potentially new therapeutic insights into the mechanisms of diseases and cancers.

## Methods

### MAGeCK-MLE: a maximum likelihood approach for essential gene detection

#### The Negative Binomial model for high-throughput CRISPR screening read counts

After read mapping, the sequencing results of CRISPR screening are presented as a read count table, where rows correspond to sgRNAs and columns correspond to samples. Read counts generated from high-throughput sequencing data have higher variances when a high number of read counts are observed (also called ‘over-dispersion’). This is usually modeled using Negative Binomial (NB) distribution, such as in the statistical models used in many RNA-seq differential expression analysis algorithms: edgeR, DESeq/DESeq2, and so on [35–37]. MAGeCK-MLE uses a similar model; briefly, the read count of sgRNA *i* in sample *j*, or *x*_*ij*_, is modeled as:

$$ {x}_{ij}\sim NB\left({\mu}_{ij},{\alpha}_i\right) $$

Where *μ*_*ij*_ and *α*_*i*_ are the mean and over-dispersion factor of the NB distribution, respectively. The mean value *μ*_*ij*_ is further modeled as:

1 $$ {\mu}_{ij}={s}_j{q}_{ij} $$

Where *s*_*j*_ is the size factor of sample *j* for adjusting sequencing depths of the samples, and q_ij_ is a variable modeling the behavior of sgRNA *i* in sample *j* that will be discussed in later sections. *s*_*j*_ is calculated by the ‘median ratio method’ in MAGeCK and DESeq2 [10, 37]:

$$ {s}_j= media{n}_i\left\{\frac{x_{ij}}{{\hat{x}}_{i\cdot }}\right\} $$

Here, ${\widehat{x}}_i$ is the geometric mean of the read counts of sgRNA *i* across all *J* samples: ${\widehat{x}}_i={\left({\displaystyle \prod_{k=1}^J}{x}_{ik}\right)}^{1/J}.$*s*_*j*_ can also be calculated based on a set of predefined ‘control’ sgRNAs instead of all sgRNAs. This is particularly useful when a majority of the genes in the library are supposed to be essential; in such cases it is not suitable to calculate *s*_*j*_ based on all sgRNAs. Both methods are implemented in MAGeCK-VISPR and users can specify which method to use.

The over-dispersion factor *α*_*i*_ is calculated based on the regression residual and will be discussed in more details in the last Methods section.

#### Modeling sgRNA knockout efficiency and complex experimental settings

Different studies demonstrated that sgRNAs have various DNA cutting efficiencies [11, 38], but such information is not considered in most essential gene calling algorithms (including MAGeCK). In MAGeCK-MLE, we use a binary variable *π*_*i*_ to model whether sgRNA *i* is efficient or not: *π*_*i*_ = 1 corresponds to an efficient sgRNA *i* and vice versa. Since *π*_*i*_ is unknown, the probability of observing a read count *x* from *x*_*ij*_ is a mixture of two distributions:

$$ P\left({x}_{ij}=x\right)=p\left({x}_{ij}=x\Big|{\pi}_i=1\right)p\left({\pi}_i=1\right)+p\left({x}_{ij}=x\Big|{\pi}_i=0\right)p\left({\pi}_i=0\right) $$

In CRISPR screening experiments, it is common to have cells treated with different conditions. For example in melanoma activation dataset [5], cell lines underwent different sgRNA expression selection methods (cells are first selected using puromycin or zeocin), duration of treatment (3-day or 21-day treatment) and drug treatments (DMSO or PLX). For an efficient sgRNA *i* (*π*_*i*_ = 1), MAGeCK-MLE uses a generalized linear model (GLM) to model the effect of *q*_*ij*_ as a linear combination of effects from different sources:

2 $$ \begin{array}{c}\hfill P\left({x}_{ij}=x\Big|{\pi}_i=1\right)\sim NB\left(x;{s}_j{q}_{ij},{\alpha}_i\right)\hfill \\ {}\hfill log\left({q}_{ij}\right)={\beta}_{i0}+{\displaystyle \sum_r}{d}_{jr}{\beta}_{gr}\hfill \end{array} $$

Here, *β*_*i*0_ is the baseline abundance of sgRNA *i*, corresponding to its abundance in an initial state (in plasmid or day 0). *d*_*jr*_ is an element of a *design matrix* given by the user (explained later), and *β*_*gr*_ is the (unknown) coefficient that we would like to estimate.

If sgRNA *i* is inefficient (*π*_*i*_ = 0), then its read counts in all samples are not determined by any experimental conditions except the baseline abundance:

3 $$ \begin{array}{c}\hfill P\left({x}_{ij}=x\Big|{\pi}_i=0\right)\sim NB\left(x;{s}_j{q}_{ij},{\alpha}_i\right)\hfill \\ {}\hfill log{q}_{ij}={\beta}_{i0}\hfill \end{array} $$

#### The design matrix

Design matrices have been used in many gene expression analysis algorithms for modeling complex experimental designs, including LIMMA [39], VOOM [40], DESeq2 [37], and so on. The design matrix *D* models the combination of effects of different conditions. For *J* samples that are affected by *R* conditions, *D* is a *J* * *R* binary matrix with element *d*_*jr*_ = 1 if sample *j* is affected by condition *R*, and 0 otherwise. An example of the design matrix is presented in Additional file 1.

Based on the design matrix, the equations in (2) and (3) can be written in a matrix form. For a gene *g* with *N* sgRNAs in *J* samples, let ${\overrightarrow{q}}_g$ be the vector of *q* values of all sgRNAs in all samples in gene *g*:

$$ {\overrightarrow{q}}_g={\left({q}_{11},{q}_{21}, \dots, {q}_{N1},\dots, {q}_{1J},{q}_{2J},\dots, {q}_{NJ}\right)}^T $$

It can be written as:

$$ \log \left({\overrightarrow{q}}_g\right)=D\hbox{'}*{\overrightarrow{\beta}}_g $$

Where ${\overrightarrow{\beta}}_g$ is a *N* + *r* vector of *β* values in Equations (2) and (3). The first *N* elements of ${\overrightarrow{\beta}}_g$ are the baseline abundances of *N* sgRNAs, and the following *R* elements of ${\overrightarrow{\beta}}_g$ are the coefficients corresponding to *R* columns in the design matrix:

$$ {\overrightarrow{\beta}}_g={\left({\beta}_{00},{\beta}_{10},\dots,\ {\beta}_{N0},{\beta}_1,\dots, {\beta}_r\right)}^T. $$

The binary *extended design matrix D*’ is used to set up the linear relationship between ${\overrightarrow{\beta}}_g$ and ${\overrightarrow{q}}_g$, and can be derived directly from the design matrix. See Additional file 1 for the definition and an example of *D*’.

#### The EM approach

MAGeCK-MLE uses a maximum likelihood estimation (MLE) approach to find the values of ${\overrightarrow{\beta^{\ast}}}_g$ The objective function of MAGeCK-MLE is:

$$ \left({\overrightarrow{\beta}}_g^{*},{\pi_i}^{*}\right)= arg\underset{\upbeta_{\mathrm{g}},\ {\pi}_i}{max}\left({\displaystyle \sum_{\begin{array}{c}\hfill i\in g,\ \hfill \\ {}\hfill j=1,\dots J\hfill \end{array}}} \log\ p\left({x}_{ij}\right)\ \right) $$

Similar to DESeq2 [37], MAGeCK-MLE also adds a prior $p\left({\overrightarrow{\beta}}_g\right)$ that follows a normal distribution centered on zero in the objective function. Adding this prior makes sure ${\overrightarrow{\beta^{\ast}}}_g$ does not become arbitrarily large, when the sgRNA knockout efficiency is low and the differences of read counts between samples are high.

The objective function can be maximized using expectation maximization (EM). At the beginning, we have an initial guess of *p*(*π*_*i*_ = 1). Subsequently, we iteratively update the values of *p*(*π*_*i*_ = 1) and $\overrightarrow{\beta}$ in the E step and the M step, respectively.

#### The initial guess of sgRNA knockout efficiency

We demonstrated that the SSC (Spacer Scoring of CRISPR) algorithm accurately predicts sgRNA knockout efficiency from genomic sequence content [11]. For each sgRNA, SSC generates an efficiency score in the range (−2,2). We scale the score linearly to the range (0,1) as an initial guess of *p*(*π*_*i*_ = 1). If no initial estimates are given, MAGeCK-MLE starts with *p*(*π*_*i*_ = 1) = 1 for all sgRNAs.

#### The expectation step

In the E step, we re-estimate the posterior probability *p*(*π*_*i*_ = 1) and the current estimation of ${\overrightarrow{\beta}}_g$:

$$ p\left({\pi}_i=1\Big|{x}_{ij},{\overrightarrow{\beta}}_g\ \right)=\frac{{\displaystyle {\prod}_j}p\left({x}_{ij}\left|{\pi}_i=1,\ {\overrightarrow{\beta}}_g\ \right)p\left({\pi}_i=1\right|{\overrightarrow{\beta}}_g\right)}{{\displaystyle {\prod}_j}p\left({x}_{ij}\Big|{\pi}_i=1,{\overrightarrow{\beta}}_g\right)p\left({\pi}_i=1\Big|{\overrightarrow{\beta}}_g\right) + {\displaystyle {\prod}_j}p\left({x}_{ij}\left|{\pi}_i=0,\ {\overrightarrow{\beta}}_g\ \right)p\left({\pi}_i=0\right|{\overrightarrow{\beta}}_g\right)\ } $$

#### The maximization step

In the M step, we maximize the values of ${\overrightarrow{\beta}}_g$ based on the values of *p*(*π*_*i*_ = 1). To derive the formula for updating ${\overrightarrow{\beta}}_g$, we write the probability of observing a read count *x* of *x*_*ij*_ as:

$$ P\left({x}_{ij}=x\right)=P{\left({x}_{ij}=x\Big|{\pi}_i=1\right)}^{I\left({\pi}_i=1\right)}*P{\left({x}_{ij}=x\Big|{\pi}_i=0\right)}^{I\left({\pi}_i=0\right)} $$

where *I*(.) is an indicator function. Taking the logarithm on both sides of the equation, we get

$$ \log P\left({x}_{ij}=x\right)=I\left({\pi}_i=1\right) \log P\left({x}_{ij}=x\Big|{\pi}_i=1\right)+I\left({\pi}_i=0\right) \log P\left({x}_{ij}=x\Big|{\pi}_i=0\right) $$

In the EM algorithm, it can be approximated by replacing the indicator function *I*(*π*_*i*_ = 1) and *I*(*π*_*i*_ = 0) with the posterior probability of *P*(*π*_*i*_ = 1) and *P*(*π*_*i*_ = 0), respectively [41], using the results from the E step. Therefore, the log likelihood function from the mixture model can be written as:

$$ \begin{array}{l}{\displaystyle \sum_{i,j}} \log P\left({x}_{ij}=x\right)\hfill \\ {}={\displaystyle {\sum}_{i,j}P\left({\pi}_i=1\Big|{x}_{ij},{\overrightarrow{\beta}}_g\right) logP\left({x}_{ij}=x\Big|\ {\pi}_i=1\right)+P\left({\pi}_i=0\Big|{x}_{ij},{\overrightarrow{\beta}}_g\right) logP\left({x}_{ij}=x\Big|{\pi}_i=0\right)}\hfill \end{array} $$

Since NB distribution belongs to exponential family distributions, a fast algorithm exists for the maximum likelihood estimation of generalized linear models [42]. Taking the prior of ${\overrightarrow{\beta}}_g$ into consideration, the objective function can be maximized using iteratively reweighted ridge regression, or weighted updates, the same the algorithm used in DESeq2 [37]. The update rule for calculating ${\overrightarrow{\beta^t}}_g$ at step *t* of the iteration can be written as:

$$ \overrightarrow{\beta_g^t}={\left({\mathrm{D}}^{\hbox{'}\mathrm{T}}W\mathrm{D}\hbox{'}+\lambda I\right)}^{-1}{\mathrm{D}}^{\hbox{'}\mathrm{T}}W\overrightarrow{z^t} $$

Here, *W* is the diagonal matrix with its values given by *w*_*ii*_ = *e*_*i*_^*t*^/(1/*μ*_*i*_ + *α*_*i*_), where *e*_*i*_^*t*^ is the current estimate of the efficiency of sgRNA $i:{e}_i^t=P\left({\pi}_i=1\Big|{x}_{ij},\overrightarrow{\beta_g^{t-1}}\right)$, *λ* is the regularization parameter in the ridge regression, and *μ*_*i*_ is the current estimate of the mean of the NB variable:

4 $$ \begin{array}{c}\kern1em \overrightarrow{\mu^t}={s}_j exp\left(\overrightarrow{h^t}\right)\kern1em \\ {}\kern1em \overrightarrow{{\mathrm{h}}^{\mathrm{t}-1}}=\mathrm{D}\hbox{'}\overrightarrow{\beta_g^{t-1}}\kern1em \end{array} $$

${\overrightarrow{\mathrm{z}}}^t$ is the residue vector of the current estimate, with its *i*th element:

$$ {z}_i^t={\mathrm{h}}_{\mathrm{i}}^{t-1}+{e}_i^t\left({\mathrm{x}}_{\mathrm{i}}-{\mu}_i^t\right)/{\mu}_i^t $$

Here, *x*_*i*_ is the read count of sgRNA *i*.

#### Convergence

The EM approach iterates the E step and the M step until it converges or reaches a predefined maximum number of iteration.

### Statistical significance

The statistical significance of ${\overrightarrow{\beta}}_g$ is calculated in both permutation and Wald test. In permutation test, MAGeCK-MLE shuffles all sgRNAs in a gene to generate empirical null distribution of ${\overrightarrow{\beta}}_g$ . The number of shufflings is a parameter specified by the user, and the default value is set to be 2*(total number of genes). In the Wald test, MAGeCK-MLE compares the value of ${\overrightarrow{\beta}}_g/SE\left({\overrightarrow{\beta}}_g\right)$ to the standard Normal distribution, where $SE\left({\overrightarrow{\beta}}_g\right)$ is the standard error of ${\overrightarrow{\beta}}_g$:

$$ \begin{array}{c}\hfill SE\left({\overrightarrow{\beta}}_g\right)=\sqrt{diag\Big(Cov\left(\left({\overrightarrow{\beta}}_g\right)\right)\ }\hfill \\ {}\hfill Cov\left(\left({\overrightarrow{\beta}}_g\right)\right)={\left({D}^{\hbox{'}T}W{D}^{\hbox{'}}+\lambda I\right)}^{-1}\left({D}^{\hbox{'}T}W{D}^{\hbox{'}}\right){\left({D}^{\hbox{'}T}W{D}^{\hbox{'}}+\lambda I\right)}^{-1}\hfill \end{array} $$

Here, $diag\left(Cov\left({\overrightarrow{\beta}}_g\right)\right)$ are the diagonal elements of the covariance matrix of ${\overrightarrow{\beta}}_g$.

### Calculating the over-dispersion factor

The over-dispersion factor, *α*_*i*_, is calculated based on the mean and variance estimation algorithm used in MAGeCK [10] and VOOM [40]. We first calculate the fitted values of ${\overrightarrow{\beta}}_g$, or ${\widehat{\beta}}_g$, using the EM algorithm proposed before, with the over-dispersion factor set to a fixed value (for example, 0.01). Then the fitted means ${\widehat{\mu}}_i$ are calculated using Equation (4), and the residual variances are calculated using the following equation:

$$ {{\widehat{\sigma}}_i}^2={\left({x}_i-{\widehat{\mu}}_i\right)}^2 $$

MAGeCK-MLE then models the sample residual variance ${\widehat{\sigma}}^2$ and fitted mean $\widehat{\mu}$ using the same model as in MAGeCK [10]:

$$ {\widehat{\sigma}}^2=\widehat{\mu}+k{\widehat{\mu}}^b $$

Where *k* and *b* are learned from the fitted means and residual variances of all sgRNA read counts. The values of *α*_*i*_ are then calculated based on the fitted values of sample residual variance ${{\widehat{\sigma}}_f}^2$ from this model:

$$ {\alpha}_i=\frac{{\widehat{\sigma}}_f^2-{\widehat{\mu}}_i}{{{\widehat{\mu}}_i}^2} $$

### Availability

The MAGeCK-VISPR workflow is available open source at http://bitbucket.org/liulab/mageck-vispr under the MIT license.

## Additional file

Additional file 1: **Supplementary materials.** (PDF 2045 kb)

## Abbreviations

AML: acute myeloid leukemia
CDF: cumulative distribution function
CML: chronic myeloid leukemia
CRISPR: clustered regularly interspaced short palindromic repeats
D3: Data-Driven Documents
dCas9: dead Cas9
DMSO: dimethyl sulfoxide
EM: expectation-maximization
GeCKO: genome-scale CRISPR/Cas9 knockout
GLM: generalized linear model
GO: gene ontology
MAGeCK: Model-based Analysis of Genome-wide CRISPR/Cas9 Knockout
MLE: maximum-likelihood estimation
NB: negative binomial
NGS: next-generation sequencing
PCA: principle component analysis
QC: quality control
RNAi: RNA interference
RRA: robust rank aggregation
SAM: Synergistic Activation Mediator
sgRNA: single-guide RNA
VISPR: VISualization of crisPR screens
