# Supplementary material for: Quality control, modeling, and visualization of CRISPR screens with MAGeCK-VISPR
Source: Genome Biol. 2015 Dec 16;16:281. doi: 10.1186/s13059-015-0843-6 (PMC4699372; doi:10.1186/s13059-015-0843-6)
Supplement: Supplementary file 1 — Supplementary materials. (PDF 2045 kb) [file 13059_2015_843_MOESM1_ESM.pdf]

Supplementary Materials

A. Comparing MAGeCK-MLE with MAGeCK-RRA on identifying known essential genes

We compared the performance of MAGeCK-RRA and MAGeCK-MLE on identifying known essential genes on from the four public datasets. For evaluation, GOrilla Gene Ontology (GO) analysis was used to evaluate the statistical significance of enriched GO terms [1]. In all datasets, both MAGeCK-MLE and MAGeCK-RRA identified essential genes that are enriched in negative selection experiments, including ribosomal subunits, spliceosome complex, and ribonucleoprotein complex (see Figure M1 and Figure M2).

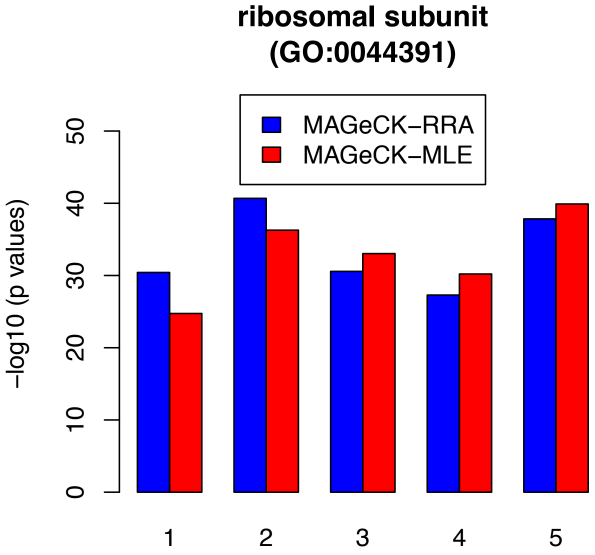

Figure M1: Ribosomal subunits are enriched in different negative selection experiments. GORilla analysis is used for the Gene Ontology (GO) analysis, and the comparisons are listed as follows: (1) CRISPR treated HL60 cells vs. day 0 in leukemia dataset; (2) CRISPR treated KBM7 cells vs. day 0 in leukemia dataset; (3) DMSO treated A375 cells in day 14 vs. plasmid in melanoma knockout dataset; (4) DMSO treated A375 cells in day 7 vs. plasmid in melanoma knockout dataset; and (5) CRISPR treated ESC cells vs. day 0 in ESC dataset.

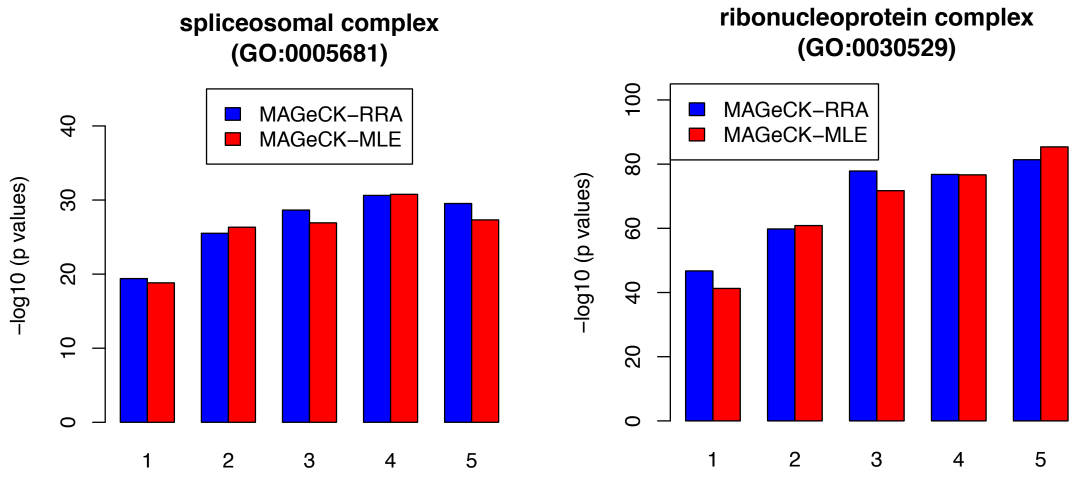

Figure M2: Spliceosome complex (left) and ribonucleoprotein complex (right) are enriched in different negative selection experiments. For the meanings of comparisons 1-5, see Figure M1.

In the melanoma knockout dataset [2], known genes related to PLX resistance are also identified in both MAGeCK-RRA and MAGeCK-MLE, including NF1, NF2, MED12, CUL3 (Table M1). In the melanoma activation dataset [3], both methods identified known genes whose over-expression contribute to faster cell growth in the PLX treated condition, including EGFR, GPR35, and LPAR1/5 (Table M2).

Genes	β score (MAGeCK-MLE)	Rank (MAGeCK-MLE)	RRA score (MAGeCK-RRA)	Rank (MAGeCK-RRA)
NF1	8.01	1	2.27e-6	9
NF2	7.00	3	4.63e-11	2
CUL3	5.60	16	1.04e-8	4
MED12	6.03	10	6.30e-12	1

Table M1: The scores and ranks of known genes whose knockout leads to PLX resistance in melanoma knockout dataset. Here, the scores and rankings of 14-day PLX treated samples vs. DMSO treated samples are shown.

Genes	beta score (MAGeCK-MLE)	Rank (MAGeCK-MLE)	RRA score (MAGeCK-RRA)	Rank (MAGeCK-RRA)
EGFR	10.36	1	2.86e-11	1
GPR35	7.76	8	3.04e-4	41
LPAR1	8.79	2	1.07e-4	25
LPAR5	8.05	4	2.54e-10	3
P2RY8	7.85	5	3.74e-11	2

Table M2: The scores and ranks of known genes whose over-expression leads to PLX resistance in melanoma activation dataset. Here, the scores and rankings of puromycin selected 21-day PLX treated samples vs. DMSO treated samples are shown.

B. Comparing MAGeCK-MLE with other methods in two-condition comparisons

We also compared MAGeCK-MLE with MAGeCK-RRA and other published algorithms, including RIGER [4] and RSA [5]. To evaluate the performance, we adopted a set of "gold-

standard” essential and non-essential genes from multiple RNAi screens [6], and calculate the precision and recall for the outputs of different algorithms. Precision and recall are defined as $TP/(TP+FP)$ and $TP/(TP+FN)$, respectively, where TP (True Positive) is the number of essential genes identified by the algorithm, FP (False Positive) is the number of non-essential genes identified by the algorithm, and FN (False Negative) is the number of essential genes not identified by the algorithm.

We compared the precision-recall curve of each algorithm on leukemia and melanoma knockout dataset, as well as the value of AUCPR (Area Under the Precision-Recall Curve) for each algorithm in Figure M**3**. For two-condition comparisons, MAGeCK-RRA, MAGeCK-MLE and RSA reached similar performances in both datasets.

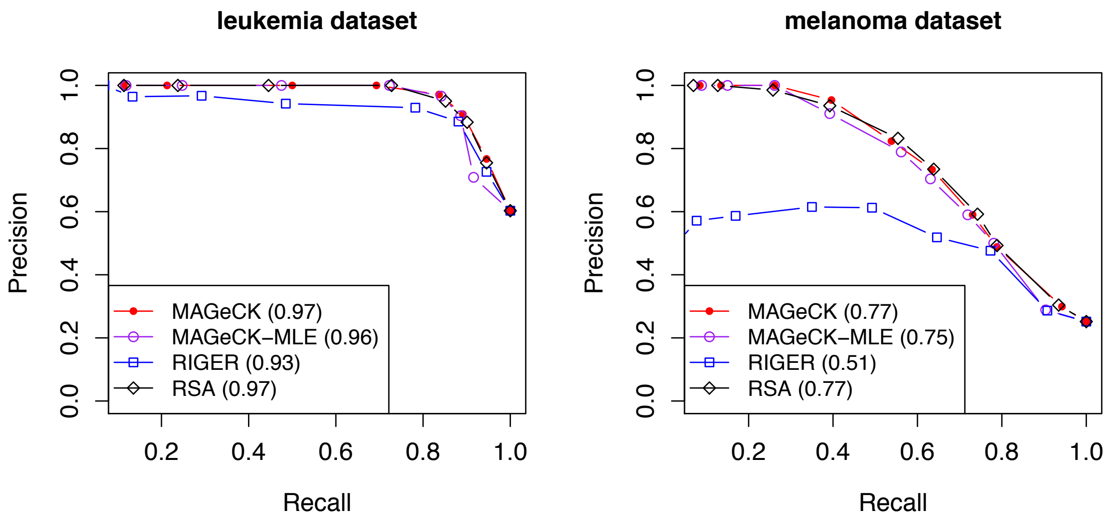

Figure M3: The Precision-Recall curve of all algorithms for leukemia dataset (left) as well as melanoma knockout dataset (right). For the ranked list of genes generated by each algorithm, “gold-standard” essential and non-essential genes from RNAi screens [6] are used calculate the values of precision and recall. The Area Under the Precision-Recall Curve value (AUCPR) for each algorithm is also displayed in the legend.

The melanoma knockout dataset includes two time point measurements (day 7 and day 14) for each condition (PLX or DMSO treatment). We used this dataset to evaluate the consistency of the results between two time points. For each algorithm and each condition, we defined a set of “reference” genes as those that are consistently ranked top on two time points, and used them to calculate the true positive rate and false positive rate for each algorithm. If the top K genes identified by each algorithm includes n “reference” genes, then the false positive rate is defined as $(K-n)/K$, and true positive rate is defined as n/M, where M is the number of “reference” genes.

We compared the Receiver Operating Characteristics (ROC) curve, as well as the Area Under the Curve (AUC) value of different algorithms in Figure M**4**. MAGeCK-MLE has a better performance compared with other algorithms, indicating that the results of MAGeCK-MLE are more consistent across similar conditions compared with other methods. This may be due to the ability of MAGeCK-MLE to model multiple conditions concurrently.

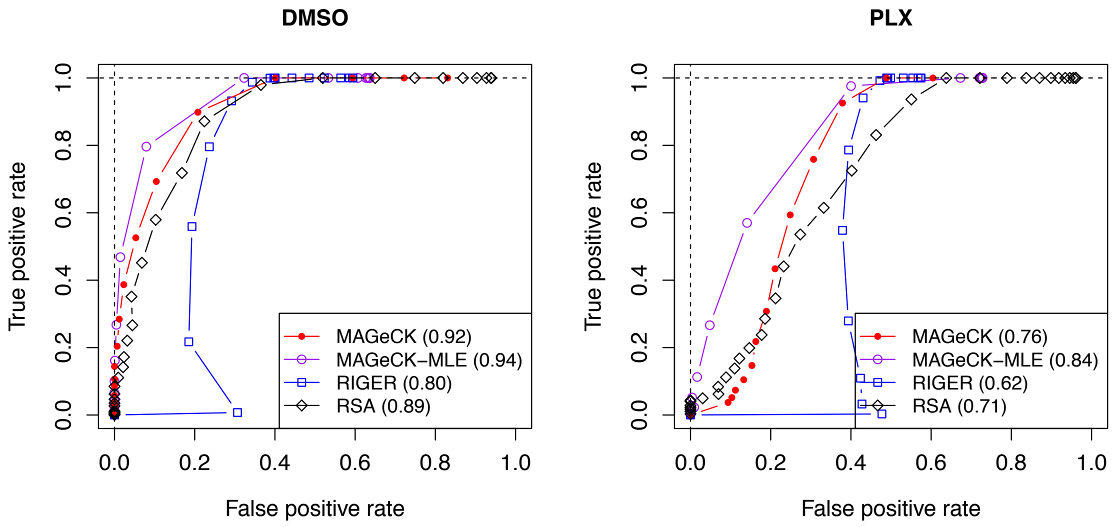

Figure M4: The ROC (Receiver Operation Characteristics) curve of two conditions (DMSO and PLX treatment) of different algorithms using melanoma knockout dataset. The "gold-standard" genes are defined separately for each method as those genes that are consistently identified as essential in two time points (7 day and 14 day). The Area Under the Curve value (AUC) for each algorithm is displayed in the legend.

C. Comparing MAGeCK-MLE with other methods in multiple conditions

Although MAGeCK-MLE is specifically designed for multiple-condition screens, other algorithms based on two-condition comparisons can also be used for multiple-condition comparisons. For these algorithms, a straightforward approach is to simply combine and compare the proper "scores" generated from two-condition comparisons.

To evaluate the performance of this approach compared with MAGeCK-MLE, we compared the scores generated by MAGeCK-RRA, RIGER and RSA in melanoma knockout dataset. For each of the four conditions (DMSO 7-day, DMSO 14-day, PLX 7-day, PLX 14-day), we compared it with the plasmid control sample and clustered the top-ranked scores across 4 conditions in Figure M5 and Figure M6, similar to what we did in Figure 4A. For RIGER, we compared the "Normalized Enrichment Scores" (NES) across different conditions. For MAGeCK-RRA and RIGER, we compared the log of RRA score and the log of p values, respectively, in both negative and positive selected genes across different conditions.

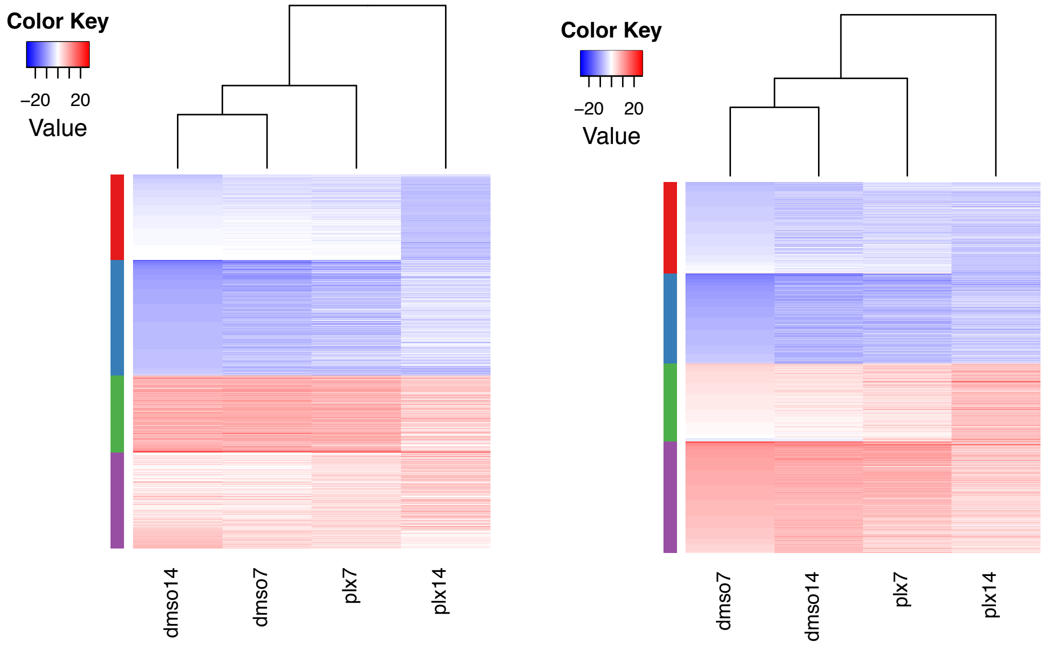

Figure M5. Top genes in DMSO14 and PLX14 conditions identified by MAGeCK-RRA (left) and RSA (right). The log(RRA score) and log(p value) are used for comparison of negatively selected genes in MAGeCK-RRA and RSA, respectively. For positive selected genes, -log(RRA score) and –log(p value) are used instead.

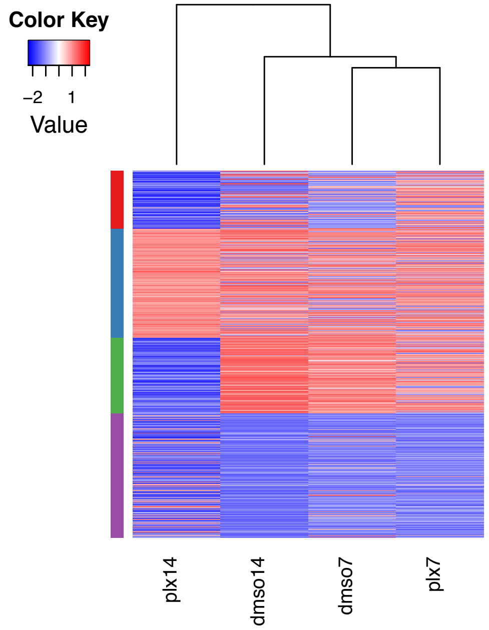

Figure M6. Top genes in DMSO14 and PLX 14 conditions identified by RIGER. The "Normalized Enrichment Score" (NES) is used for comparing multiple conditions.

MAGeCK-RRA, RIGER and RSA identified genes that are similar to some patterns displaced in Figure 4A, including genes that are negatively selected in PLX or DMSO conditions. However, a key difference is that genes that are strongly positively selected in PLX14 condition (cluster 4 in Figure 4A) are not identified by MAGeCK-RRA, RIGER or RSA in Figure M5 and

Figure M6. These genes include the known and validated genes in the original study: NF1, NF2, MED12, CUL3, *etc.* [2]. For these algorithms, the scores of these genes can still reflect the stronger selection in PLX 14-day condition, but the distributions of scores are similar across conditions (Figure 4B). In contrast, MAGeCK-MLE identified a set of genes whose scores are much higher than any other genes in other conditions (Figure 4B) -- an indication that these genes are under strongly positive selection in PLX 14-day condition.

D. sgRNA efficiency estimation improves gene identification

We investigate whether the information of sgRNA efficiency can improve the performance of gene identification. To do this, we ran MAGeCK-MLE on two modes, one with sgRNA efficiency estimation from the SSC algorithm [7] and iteratively update sgRNA efficiency during the EM algorithm, and the other assuming all sgRNAs are efficient. The following tables (Table M3 and Table M4) demonstrate the improvement of identifying essential genes using sgRNA efficiency estimation.

GO Terms	p-value (without sgRNA efficiency estimation)	p-value (with sgRNA efficiency estimation)
GO:0044391 ribosomal subunit	7.44e-32	1.84e-37
GO:0005681 spliceosomal complex	4.66e-22	3.31e-23
GO:0030529 ribonucleoprotein complex	2.42e-49	2.39e-50

Table M3: The p values of enrichment in identifying essential genes of HL60 with or without sgRNA efficiency estimation in leukemia dataset.

GO Terms	p-value (without sgRNA efficiency estimation)	p-value (with sgRNA efficiency estimation)
GO:0044391 ribosomal subunit	5.64e-42	5.08e-49
GO:0005681 spliceosomal complex	1.81e-26	1.91e-31
GO:0030529 ribonucleoprotein complex	2.85e-68	1.04-72

Table M4: The p values of enrichment in identifying essential genes of KBM7 with or without sgRNA efficiency estimation in leukemia dataset.

We also plotted the Precision-Recall curve (PR curve) of two modes using the “gold-standard” essential and non-essential genes, similar to the approach we performed in Section B (Figure M**7**). The value of AUCPR (Area Under the Precision-Recall Curve) using sgRNA efficiency

estimation is higher than that without such information, demonstrating that it better improves the performance of gene callings.

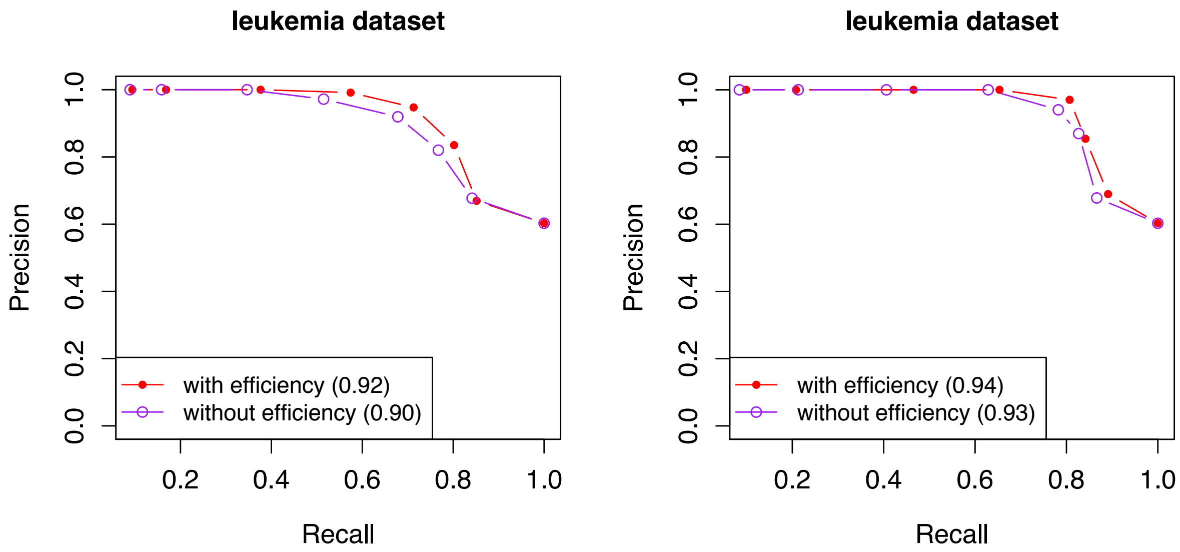

Figure M7: The Precision-Recall curve of MAGeCK-MLE with or without sgRNA efficiency in leukemia dataset, including the estimation of HL60 essential genes (left) and KBM7 essential genes (right). We used “gold-standard” essential and non-essential genes from RNAi screens [6] to calculate the values of precision and recall. The Area Under the Precision-Recall Curve value (AUCPR) for each algorithm is also displayed in the legend.

E. The design matrix and extended design matrix

In MAGeCK-MLE, we use a *design matrix* to model complex experimental conditions. Using an extended design matrix, the q values in the Methods section can be written conveniently in a matrix form. The *extended design matrix* D' has $N * J$ rows and $N + r$ columns, and can be derived from the design matrix.

For a given design matrix D with size $J * R$, the extended design matrix can be written in the following form:

Rows/Columns	1..N	$N+1$	$N+2$	…	$N+R$
1 … N (sample1)	I_N	d_{11}	d_{12}	…	d_{1R}
N+1 … 2N (sample 2)	I_N	d_{21}	d_{22}	…	d_{2R}
…	I_N	…	…	…	…

...	I_N	...	...	...	...
(J-1)*N- J*N (sample J)	I_N	d_{J1}	d_{J2}	...	d_{JR}

Table M5: The extended design matrix.

where I_N is the identity matrix with size N, and d_{ij} is the element of row i and column j in D.

Under the mixture model, the extended design matrix in Table M5 assumes all sgRNAs are efficient ($\pi_i = 1$). If the sgRNAs are not efficient, the extended design matrix becomes

Rows/Columns	1..N	$N+1$	$N+2$	...	$N+R$
1 … N (sample1)	I_N	0	0	...	0
N+1 … 2N (sample 2)	I_N	0	0	...	0
...	I_N	...	...	...	...
...	I_N	...	...	...	...
(J-1)*N- J*N (sample J)	I_N	0	0	...	0

Table M6: The extended design matrix with inefficient sgRNAs.

An example of the design matrix and extended design matrix is given below. Suppose we have 5 samples: sample 0 is the baseline sample (*e.g.,* plasmid), sample 1 and 2 are from cell line A, and sample 2 are from cell line B. Sample 1 and 3 are treated with drug X, while sample 2 and 4 are treated with drug Y. In this example, three factors should be considered: baseline factor (β_0), the cell-line specific factor for cell line A and B (β_1, β_2) and the drug specific factor (β_3, β_4). The design matrix (5*5) can be written as

Samples	β_0	β_1	β_2	β_3	β_4
1	1	0	0	0	0
2	1	1	0	1	0
3	1	1	0	0	1
4	1	0	1	1	0

5	1	0	1	0	1

In the above example, if one gene has 3 efficient sgRNAs (N=3), the extended design matrix becomes:

Samples	β_{00} (sgRNA 0)	β_{01} (sgRNA 1)	β_{02} (sgRNA 2)	β_1	β_2	β_3	β_4
1	1			0	0	0	0
		1		0	0	0	0
			1	0	0	0	0
2	1			1	0	1	0
		1		1	0	1	0
			1	1	0	1	0
3	1			1	0	0	1
		1		1	0	0	1
			1	1	0	0	1
4	1			0	1	1	0
		1		0	1	1	0
			1	0	1	1	0
5	1			0	1	0	1
		1		0	1	0	1
			1	0	1	0	1

If all sgRNAs are inefficient, the extended design matrix becomes:

Samples	β_{00} (sgRNA 0)	β_{01} (sgRNA 1)	β_{02} (sgRNA 2)	β_1	β_2	β_3	β_4
1	1			0	0	0	0
		1		0	0	0	0
			1	0	0	0	0
2	1			0	0	0	0
		1		0	0	0	0
			1	0	0	0	0
3	1			0	0	0	0
		1		0	0	0	0
			1	0	0	0	0
4	1			0	0	1	0
		1		0	0	1	0
			1	0	0	1	0
5	1			0	0	0	0
		1		0	0	0	0
			1	0	0	0	0

F. The design matrixes of four datasets

Leukemia dataset [8] includes 4 samples: the initial state and the final state of HL60 and KBM7 cells. MAGeCK-MLE uses both initial state samples as “baseline” samples, and the design matrix is specified in Table M7.

Samples	baseline	HL60	KBM7
HL60.initial	1	0	0
KBM7.initial	1	0	0
HL60.final	1	1	0
KBM7.final	1	0	1

Table M7: The design matrix used in leukemia dataset.

ESC dataset [9] includes 3 samples, 1 plasmid, and 2 replicates of CRISPR screened ESC cells. The design matrix is as follows.

samples	baseline	esc
plasmid	1	0
esc1	1	1
esc2	1	1

Table M8: The design matrix used in ESC dataset.

Melanoma knockout dataset [2] includes 9 samples: PLX treated and DMSO treated A375 cells with two time windows (7-day and 14-day), 2 replicates for each condition, and 1 plasmid. We intend to model the effects of 4 factors, 7-day and 14-day, DMSO and PLX treatments. The design matrix is shown in Table M9.

Samples	baseline	DMSO7	DMSO14	PLX7	PLX14
plasmid	1	0	0	0	0
D7_R1	1	1	0	0	0
D7_R2	1	1	0	0	0
D14_R1	1	0	1	0	0
D14_R2	1	0	1	0	0
PLX7_R1	1	0	0	1	0
PLX7_R2	1	0	0	1	0
PLX14_R1	1	0	0	0	1
PLX14_R2	1	0	0	0	1

Table M9: The design matrix used in melanoma knockout dataset.

Melanoma activation dataset [3] includes 14 samples. Two are plasmid libraries, four are zeocin and puromycin selected samples in day 3 (2 replicates for each), eight are zeocin and puromycin selected, PLX or DMSO treated samples in day 21 (2 replicates for each). The design matrix is shown in Table M10.

Samples	baseline	zero_d3	puro_d3	zero_d21 _DMSO	zero_d 21_PLX	puro_d21 _DMSO	puro_d21 _PLX
zeo_plasmid_library	1	0	0	0	0	0	0

puro_plasmid_library	1	0	0	0	0	0	0
zeo_d3_rep_1	1	1	0	0	0	0	0
puro_d3_rep1	1	0	1	0	0	0	0
zeo_d3_rep2	1	1	0	0	0	0	0
puro_d3_rep2	1	0	1	0	0	0	0
zeo_d21_DMSO_rep1	1	0	0	1	0	0	0
puro_d21_DMSO_rep1	1	0	0	0	0	1	0
zeo_d21_DMSO_rep2	1	0	0	1	0	0	0
puro_d21_DMSO_rep2	1	0	0	0	0	1	0
zeo_d21_PLX_rep1	1	0	0	0	1	0	0
puro_d21_PLX_rep1	1	0	0	0	0	0	1
zeo_d21_PLX_rep2	1	0	0	0	1	0	0
puro_d21_PLX_rep2	1	0	0	0	0	0	1

Table M10: The design matrix used in melanoma activation dataset.

References

1. Eden E, Navon R, Steinfeld I, Lipson D, Yakhini Z: **GOrilla: a tool for discovery and visualization of enriched GO terms in ranked gene lists.** *BMC Bioinformatics* 2009, **10**:48.

2. Shalem O, Sanjana NE, Hartenian E, Shi X, Scott DA, Mikkelsen TS, Heckl D, Ebert BL, Root DE, Doench JG, Zhang F: **Genome-Scale CRISPR-Cas9 Knockout Screening in Human Cells**. *Science* 2014, **343**:84–87.

3. Konermann S, Brigham MD, Trevino AE, Joung J, Abudayyeh OO, Barcena C, Hsu PD, Habib N, Gootenberg JS, Nishimasu H, Nureki O, Zhang F: **Genome-scale transcriptional activation by an engineered CRISPR-Cas9 complex.** *Nature* 2015, **517**:583–588.

4. Luo B, Cheung HW, Subramanian A, Sharifnia T, Okamoto M, Yang X, Hinkle G, Boehm JS, Beroukhim R, Weir BA, Mermel C, Barbie DA, Awad T, Zhou X, Nguyen T, Piqani B, Li C, Golub TR, Meyerson M, Hacohen N, Hahn WC, Lander ES, Sabatini DM, Root DE: **Highly parallel identification of essential genes in cancer cells.** 2008, **105**:20380–20385.

5. König R, Chiang C-Y, Tu BP, Yan SF, DeJesus PD, Romero A, Bergauer T, Orth A, Krueger U, Zhou Y, Chanda SK: **A probability-based approach for the analysis of large-scale RNAi screens.** *Nat Methods* 2007, **4**:847–849.

6. Hart T, Brown KR, Sircoulomb F, Rottapel R, Moffat J: **Measuring error rates in genomic perturbation screens: gold standards for human functional genomics.** *Mol Syst Biol* 2014, **10**:733–733.

7. Xu H, Xiao T, Chen C-H, Li W, Meyer C, Wu Q, Wu D, Cong L, Zhang F, Liu JS, Brown M, Liu SX: **Sequence determinants of improved CRISPR sgRNA design.** *Genome Res* 2015:gr.191452.115.

8. Wang T, Wei JJ, Sabatini DM, Lander ES: **Genetic Screens in Human Cells Using the CRISPR-Cas9 System**. *Science* 2014, **343**:80–84.

9. Koike-Yusa H, Li Y, Tan E-P, Velasco-Herrera MDC, Yusa K: **Genome-wide recessive genetic screening in mammalian cells with a lentiviral CRISPR-guide RNA library.** *Nat Biotechnol* 2014, **32**:267–273.
